# Gender-based differences in the antidepressant treatment of patients with depression in German psychiatric practices

**DOI:** 10.3205/000229

**Published:** 2016-02-15

**Authors:** Louis Jacob, Karel Kostev

**Affiliations:** 1 Department of Biology, École Normale Supérieure de Lyon, Lyon, France; 2 IMS Health, Epidemiology, Frankfurt/Main, Germany

**Keywords:** depression, gender, female patients

## Abstract

**Background:** Depression is recognized as the leading cause of disability in the world. Our goal was to compare treatment initiation in men and women treated in German neuropsychiatric practices after diagnosis of depression.

**Methods:** Patients aged between 18 and 80 first diagnosed with depression between 2010 and 2013 were identified by 223 psychiatrists in the IMS Disease Analyzer database. Patients who had received antidepressant prescriptions prior to the index date were excluded. The main outcome measure was the initiation of antidepressant drug therapy in men and women within three years after index date in three subgroups of different severity (mild, moderate and severe depression).

**Results:** A total of 35,495 men and 54,467 women were included in this study. After 3 years of follow-up, 77.3% of men and 78.5% of women diagnosed with mild depression (p-value=0.887), 89.2% of men and 90.7% of women with moderate depression (p-value=0.084), and 88.6% of men and 89.5% of women with severe depression (p-value=0.769) had been treated. No association was found between the chances of treatment initiation after diagnosis of depression and gender. Finally, patients with moderate and severe depression were more likely to receive therapy than those with mild depression. Selective serotonin reuptake inhibitors and tricyclic antidepressants were the two most commonly prescribed families of drugs in this study (SSRIs: 34.5% to 44.6%, and TCAs: 19.1% to 26.9%).

**Conclusions:** Gender did not impact therapy initiation in depressed patients. Further studies are needed to identify other potential factors involved.

## Zusammenfassung

**Hintergrund:** Depression wird als die häufigste Ursache von Erwerbsunfähigkeit in der Welt anerkannt. Das Ziel dieser Studie ist es, den Behandlungsbeginn bei Männern und Frauen in der deutschen neuropsychiatrischen Praxis, welche nach der Diagnose Depression medikamentös behandelt wurden, zu vergleichen.

**Methoden:** Es wurden Patienten im Alter zwischen 18 und 80, mit erster diagnostizierter Depression (Indexdatum) zwischen 2010 und 2013 von 223 Psychiatern im IMS Disease Analyzer identifiziert. Patienten mit Antidepressiva-Verordnungen vor dem Indexdatum wurden exkludiert. Das Ziel war es, den Beginn der antidepressiven Therapie bei Männern und Frauen innerhalb der drei Jahre nach dem Indexdatum in drei Gruppen zu unterteilen (milde, mittelschwere und schwere Depression).

**Ergebnis:** 35.495 Männer und 54.467 Frauen wurden in die Studie eingeschlossen. Drei Jahre nach der Erstdiagnose Depression wurden 77,3% der Männer und 78,5% der Frauen mit milder Depression (p-Wert=0,887), 89,2% der Männer und 90,7% der Frauen mit mittelschwerer Depression (p-Wert=0,0884) und 88,6% der Männer und 89,5% der Frauen mit schwerer Depression (p-Wert=0,769) behandelt. Es wurde kein Zusammenhang zwischen dem Therapiewechsel nach Behandlungsbeginn nach der Depressionsdiagnose und dem Geschlecht gefunden. Vielmehr wurde festgestellt, dass Patienten mit mittelschwerer und schwerer Depression eher eine Therapie erhielten, als solche mit leichten Depressionen. Selektive Serotonin-Wiederaufnahme-Hemmer und trizyklische Antidepressiva waren dabei die am häufigsten verschriebenen Medikamente in dieser Studie (SSRIs: 34,5% bis 44,6%, und TCAs: 19,1% bis 26,9%).

**Schlussfolgerungen:** Das Geschlecht hat keinen Einfluss auf die Therapie bei depressiven Patienten. Weitere Studien sind erforderlich um andere mögliche Faktoren zu identifizieren.

## Introduction

Depression is recognized as the leading cause of disability in the world, affecting more than 350 million people [1]. In Europe, 7% of the population suffer from major depression each year, with this figure increasing to 25% when anxiety and other mood disorders are included [1]. Thus, this psychiatric condition has a significant impact on global health and on the economies of European countries [2].

Although people with depression usually prefer psychological and psychosocial treatments to medications [3], and although such psychological and psychosocial interventions are effective on a broad range of mood disorders [4], [5], medications are usually needed in more severe forms of the disease to optimize the benefits of the various therapies initiated by physicians. The main drugs prescribed in Europe are tricyclic antidepressants (TCAs), selective serotonin reuptake inhibitors (SSRIs), and serotonin and norepinephrine reuptake inhibitors (SNRIs) [5]. TCAs, which have been prescribed for decades and are among the earliest antidepressant molecules developed, inhibit the re-uptake of monoamine neurotransmitters in the presynaptic neuron and are thus associated with anticholinergic side effects (e.g., blurred vision, constipation and sweating) [6], [7]. Although such adverse events are less common with SSRIs [5], these other medications (i.e., fluvoxamine, fluoxetine and paroxetine) may inhibit cytochrome enzymes and may also cause severe drug interactions [8]. SNRIs, which were launched more recently in the mid-1990s, work by inhibiting the reuptake of both serotonin and norepinephrine, and are notably effective in the treatment of major depression [9], [10]. Nonetheless, SNRIs are also associated with several side effects, such as nausea, dry mouth and excessive sweating.

To date, several works have been published on gender-related differences in depression and their potential implications for treatments [11], [12], [13]. Nonetheless, little is known about how the therapies and management provided by physicians differ for men and women. Therefore, the goal of our study was to compare treatment initiation in men and women treated in German neuropsychiatric practices after initial diagnosis of depression.

## Methods

### Database

The Disease Analyzer database (IMS Health) compiles drug prescriptions, diagnoses, basic medical and demographic data obtained directly and in anonymous format from computer systems used in the practices of psychiatrists [14]. Diagnoses (ICD-10), prescriptions (Anatomical Therapeutic Chemical (ATC) classification system) and the quality of reported data are monitored by IMS based on a number of criteria (e.g., completeness of documentation, linkage between diagnoses and prescriptions).

In Germany, the sampling methods used for the selection of physicians’ practices is appropriate to obtain a representative database of neuropsychiatric practices [14], [15]. The sampling method for the Disease Analyzer database is based on summary statistics from all doctors in Germany published yearly by the German Medical Association (Bundesärztekammer, http://www.baek.de/). These statistics are used to determine the panel design according to the following strata: specialist group, German federal state, community size category, and age of physician. 

This panel design forms the basis for the acquisition of the practices processed in the Disease Analyzer. To account for natural fluctuation in the practices and an annual check of the summary statistics by the German Medical Association, the panel design is adjusted each year. The sampling plan is subdivided into 8 regions, which are summaries of the sixteen German federal states. This stratification results in 176 cells derived from the summary statistics with regard to specialist fields and proportional to the summary statistics with regard to the German federal states. 

### Study population

Patients with a first-time documentation of depression with known severity level (ICD-10: F320, F321, F322, F323, F330, F331, F332, F333) between January 2010 and December 2013 (index date) were identified by 223 psychiatrists in the IMS Disease Analyzer database. First-time documentation means that no depression diagnosis (F32, F33 including diagnoses with unknown severity) was documented in the whole patient history prior to index date). The last follow-up ended in July 2015. A total of 89,962 patients were available for analysis. Age of 18 to 80 years at the index date was applied as a further inclusion criterion. Patients who had received prescriptions for antidepressant drugs (ATC: N06A) in the whole available history prior to the index date were excluded. 

### Study outcome

The main outcome measure was the initiation of antidepressant drug therapy (ATC: N06A) in men and women within three years after index date in the three subgroups: patients with mild depressive episode (ICD-10: F320, F330), moderate depressive episode (F321, F331) and severe depressive episode (F323, F324, F333, F334). The shares of patients initially treated with SSR, SNRI, tricyclic, tetracyclic, herbal and other antidepressants were also estimated. Demographic data included sex and age.

### Statistical analyses

Descriptive analyses were obtained for demographic and clinical variables (gender, age, and severity of depressive episodes). Mean ± SD (standard deviation) were calculated for age, while proportions were calculated for other variables. Time to depression treatment in patients newly diagnosed with depression was analyzed separately for men and women using Kaplan-Meier curves. A multivariate Cox regression model was used to predict treatment initiation after diagnosis of depression on the basis of patient characteristics (gender, age and severity of depression). P values <0.05 were considered statistically significant. Analyses were carried out using SAS (statistical analysis system) version 9.3.

## Results

### Patient characteristics

Patient characteristics are shown in Table 1 (Tab. 1). A total of 35,495 men and 54,467 women were included in this study. The mean age was 48.4 years (SD=14.6) in men and 49.3 years (SD=15.4) in women (p-value<0.001). Men displayed severe depression less frequently than women (33.5% versus 35.1%, p-value<0.001), whereas they suffered more commonly from moderate depression (56.9% versus 55.5%, p-value<0.001). The prevalence of mild depression did not differ significantly between men and women (9.4% versus 9.6%, p-value=0.320).

### Shares of depressed patients receiving treatment

**Figure 1:**
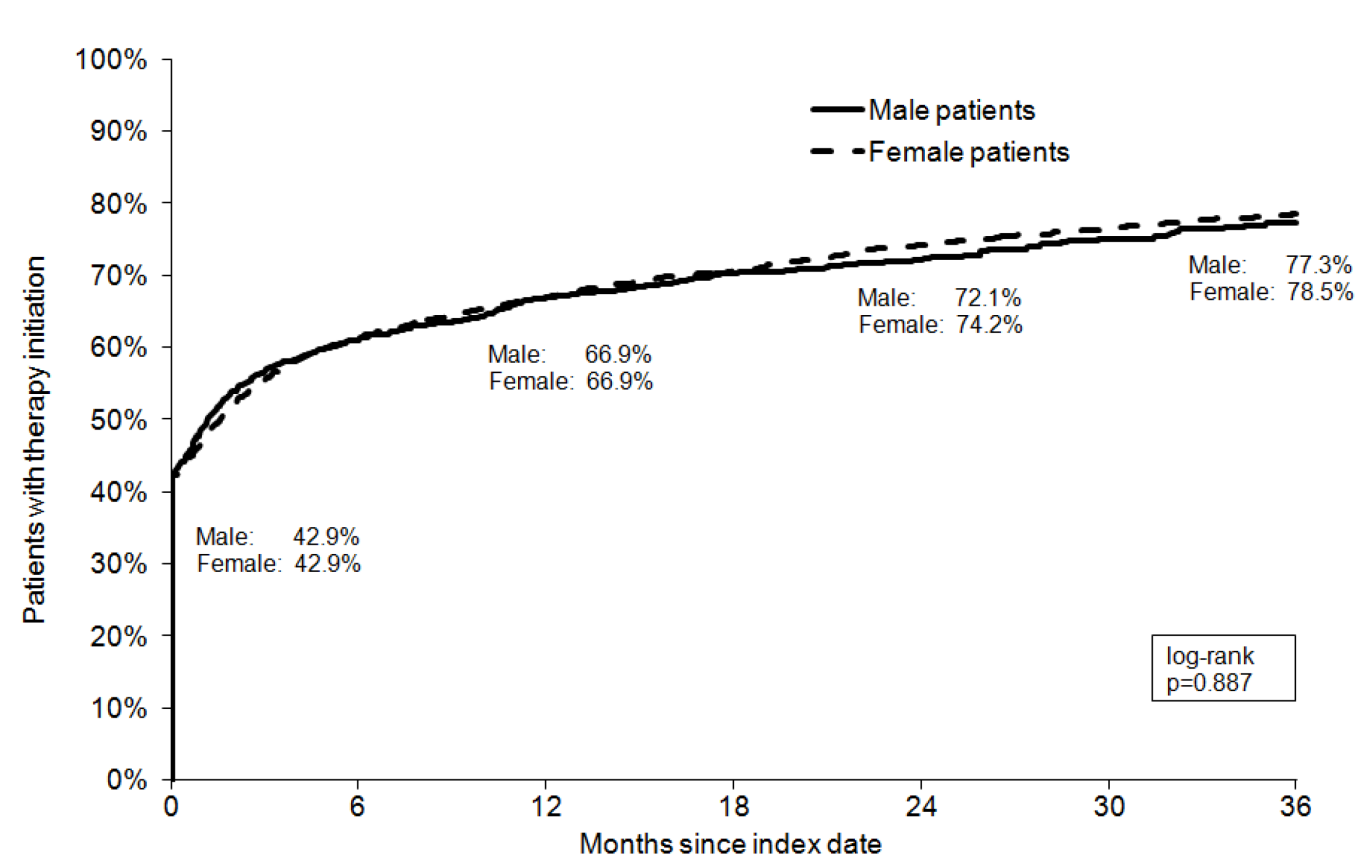
Kaplan-Meier curves for shares of patients receiving treatment for mild depression

**Figure 2:**
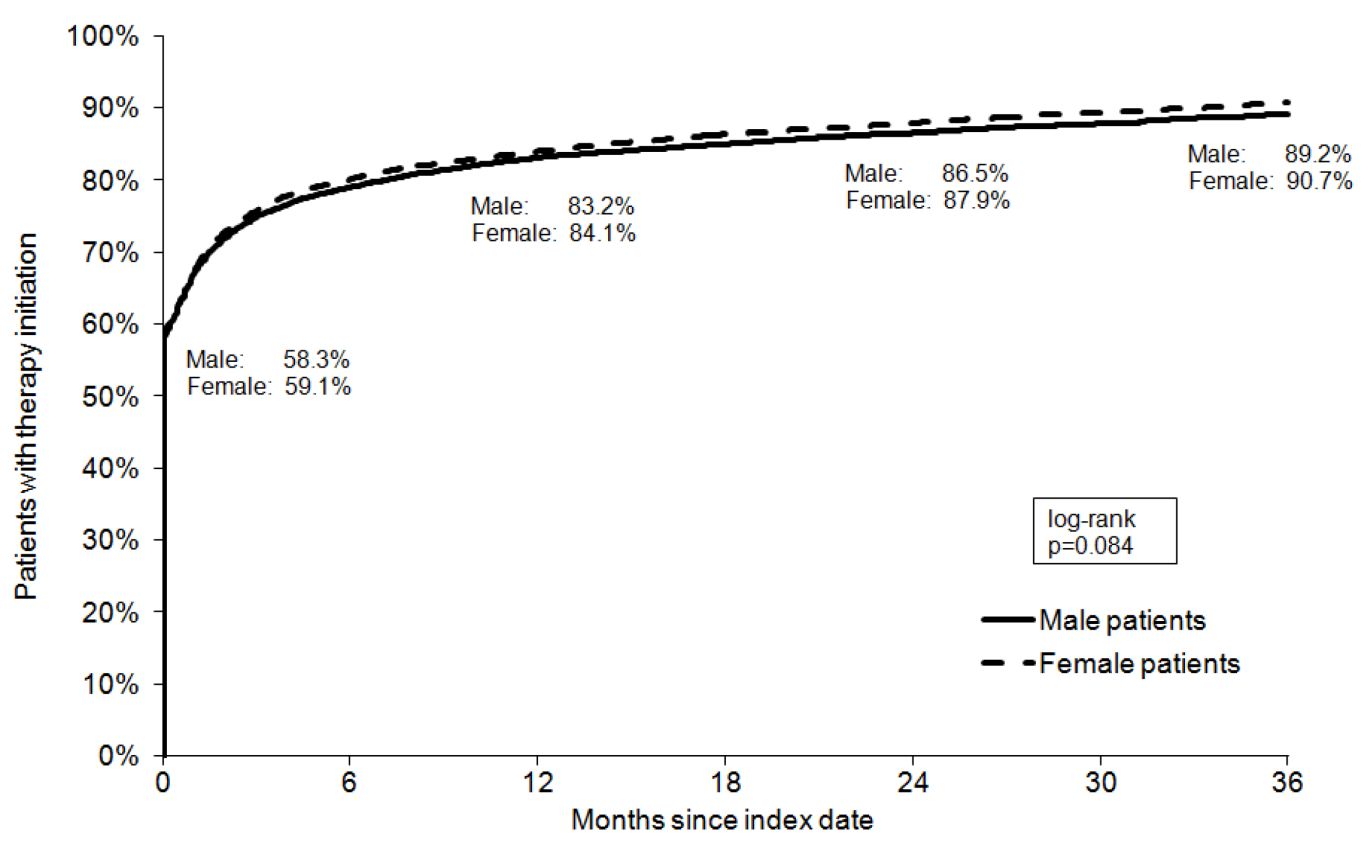
Kaplan-Meier curves for shares of patients receiving treatment for moderate depression

**Figure 3:**
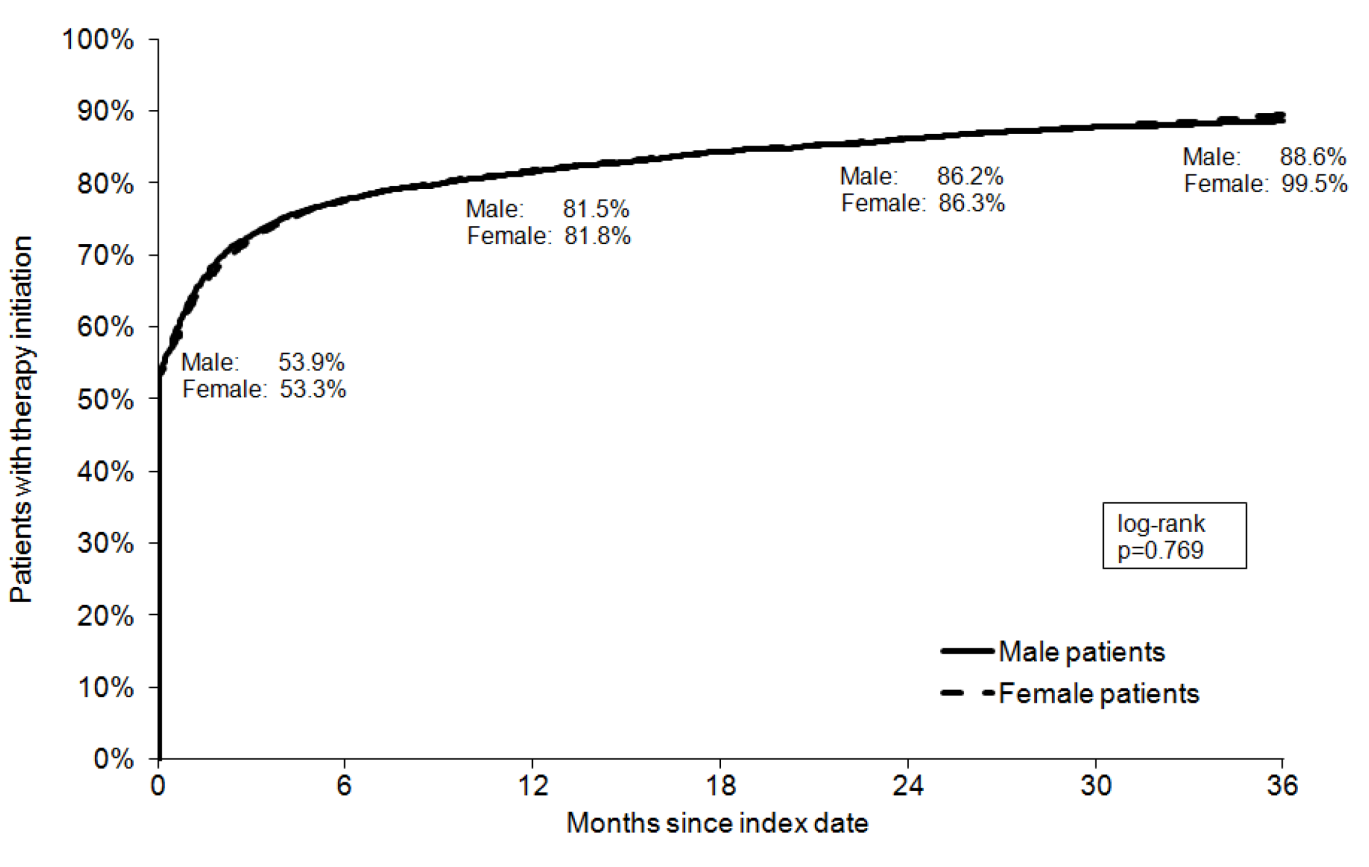
Kaplan-Meier curves for shares of patients receiving treatment for severe depression

Kaplan-Meier curves for shares of depressed men and women in the three different groups are displayed in Figure 1 (Fig. 1), Figure 2 (Fig. 2), and Figure 3 (Fig. 3). After 3 years of follow-up, 77.3% of men and 78.5% of women diagnosed with mild depression (log-rank p-value=0.887), 89.2% of men and 90.7% of women with moderate depression (log-rank p-value=0.084), and 88.6% of men and 89.5% of women with severe depression (log-rank p-value=0.769) had been treated. Table 2 (Tab. 2) shows the results of the multivariate Cox regression model. No association was found between the chances of treatment initiation after depression diagnosis and gender (OR=0.99, 95% CI: 0.98–1.01). By contrast, the chances of receiving treatment decreased slightly with age (>60 versus ≤40 years: OR=0.96, 95% CI: 0.94–0.99). Finally, patients with moderate and severe depression were more likely to receive therapy than patients with mild depression (moderate: OR=1.45, 95% CI: 1.41–1.49; and severe: OR=1.35, 95% CI: 1.31–1.39).

### Therapy classes

Table 3 (Tab. 3) indicates the proportions of the different antidepressant classes prescribed by German psychiatrists. SSRIs and TCAs were the two most commonly prescribed families of drugs in this study (SSRIs: 34.5% to 44.6%, and TCAs: 19.1% to 26.9% of patients). In cases of mild depression, SSRIs were more commonly prescribed in men than in women (44.6% versus 41.1%, p-value=0.012), whereas TCAs were more frequently prescribed in women than in men (26.9% versus 22.5%, p-value<0.001). In cases of moderate depression, SSRIs, TCAs and tetracyclic antidepressants were more commonly prescribed in men than in women (43.4% versus 41.7%, 20.8% versus 19.1%, and 18.7% versus 14.0%, p-values<0.001). In patients with severe depression, tetracyclic antidepressants were more commonly prescribed in men than in women (21.0% versus 17.1%, p-value=0.012), whereas SNRIs were more frequently administered in women than in men (15.3% versus 13.7%, p-value=0.001).

## Discussion

In our study, we demonstrated that the chances of therapy initiation after depression diagnosis were not associated with gender to any significant extent. Furthermore, we found that people with moderate and severe depression were more likely to receive therapy than those with mild depression. Finally, we showed that SSRIs and TCAs were the two families of drugs prescribed most commonly by German psychiatrists. The use of SSRIs was more frequent in men than in women with mild and moderate depression. By contrast, the use of TCAs was less common in men with mild depression and more common in men with moderate depression, than in women with mild and moderate depression respectively. Before going further, it is important to remind that our article only compared treatment initiation in men and women treated in German neuropsychiatric practices after diagnosis of depression. Thus, we do not discuss the use of pharmacotherapy in depressed patients. 

With hundreds of millions of people affected worldwide, depression is the leading global cause of disability [1]. It has recently been shown that this psychiatric condition has a significant impact on healthcare costs in Europe, and more particularly in Germany [16], [2]. In 2014, a study conducted in eight different German cities and including 1,050 randomly selected multimorbid primary care patients aged 65 to 85 demonstrated that the prevalence of depression was 10.7% and that this disorder led to a 2.5-fold increase in the mean total health-related costs per six-month period [16]. Thus, there is a need for early diagnosis, treatment and management of people with depression. 

Although numerous psychological, psychosocial and medical therapies are available nowadays for the treatment of depression, only 50% of patients suffering from major depressive episodes are treated by their physicians and monitored by other health professionals [1]. In 1994, Coryell and colleagues demonstrated in 547 individuals who had suffered episodes of major depression (313 treated and 234 untreated individuals) that treated patients were significantly older and were more likely to be married than untreated patients [17]. By contrast, the authors found that the two groups did not differ for gender, educational level, household income or religious preference [17]. Four years later, Angst decided to focus on the hypothetical overrepresentation of women among treated cases of major depressive episodes, and showed in 591 depressed patients from Zurich that the proportion of women in the treated population exceeded 80%, whereas the proportion of women in the untreated population was 55% [18]. Moreover, those who were treated were also more severely depressed than the others. Interestingly, a family history of depression and the age of onset were not associated with treatment initiation to a significant extent [18]. Therefore, these findings suggest that gender and the severity of the disease may impact a person’s chances of receiving therapy, as men and patients with mild depression received treatment less frequently than women and patients with moderate or severe depression. Although we found that the severity of depression was positively associated with the chances of receiving treatment, our study showed, in line with the work of Coryell and colleagues [17], that gender did not significantly impact therapy initiation. There are several hypotheses that may explain this difference between the results of Angst and our own results. Firstly, it is important to remember that the previous author only compared treated and untreated populations, and did not use regression models to predict treatment initiation after diagnosis of depression based on patient characteristics. Secondly, he included 591 depressed patients aged between 20 and 35 from Switzerland, whereas we selected 89,962 patients aged between 18 and 80 from German neuropsychiatric practices. Thus, when considering younger populations, it is possible that depressed women are more likely to initiate therapy than depressed men. Nevertheless, when studying broader age ranges, we did not find that gender had any significant impact, similarly to Coryell et al., who selected patients aged over 17 [17].

Finally, we found that SSRIs and TCAs were the two most frequently used antidepressants, and these results are in line with the existing literature. Indeed, in 2008, Bauer and colleagues analyzed antidepressant prescribing patterns in 12 European countries and showed that SSRIs were the most commonly prescribed drugs [19]. Interestingly, the use of TCAs was also dominant in Germany, contrary to other countries, where these drugs were usually used less frequently than SNRIs. Furthermore, we also found some differences in prescribing patterns for men and women in the three subgroups (mild, moderate and severe depression). Although most of these differences were small, it is still possible that they may be clinically relevant. 

This study was subject to several limitations. First of all, diagnoses were only established by psychiatrists. Secondly, no data were available on socioeconomic status and quality of life (e.g., marital status, alcohol/drug abuse and stressful experiences), although socioeconomic factors might also be predictors of treatment initiation. Moreover, no information was available about psychotherapy, which is a further option for the treatment of depression and has an impact on antidepressant medication. Finally, the strength of the study is the large nationwide database and the unbiased assessment of diagnoses. However, having a large sample size, very small differences can be detected as significant and it should be noted by the interpretation of results.

Overall, this study shows that gender does not impact treatment initiation in depressed patients, suggesting that men and women are treated equally by German psychiatrists. Further studies are needed to identify other potential factors associated with the chances of therapy initiation.

## Notes

### Competing interests

Karel Kostev is an employee of IMS Health. IMS Health (http://www.imshealth.de/sites/en/about-us/our-company) is a commercial research institute providing information, services and technology for the healthcare industry. Louis Jacob declares that he has no competing interests.

## Figures and Tables

**Table 1:**
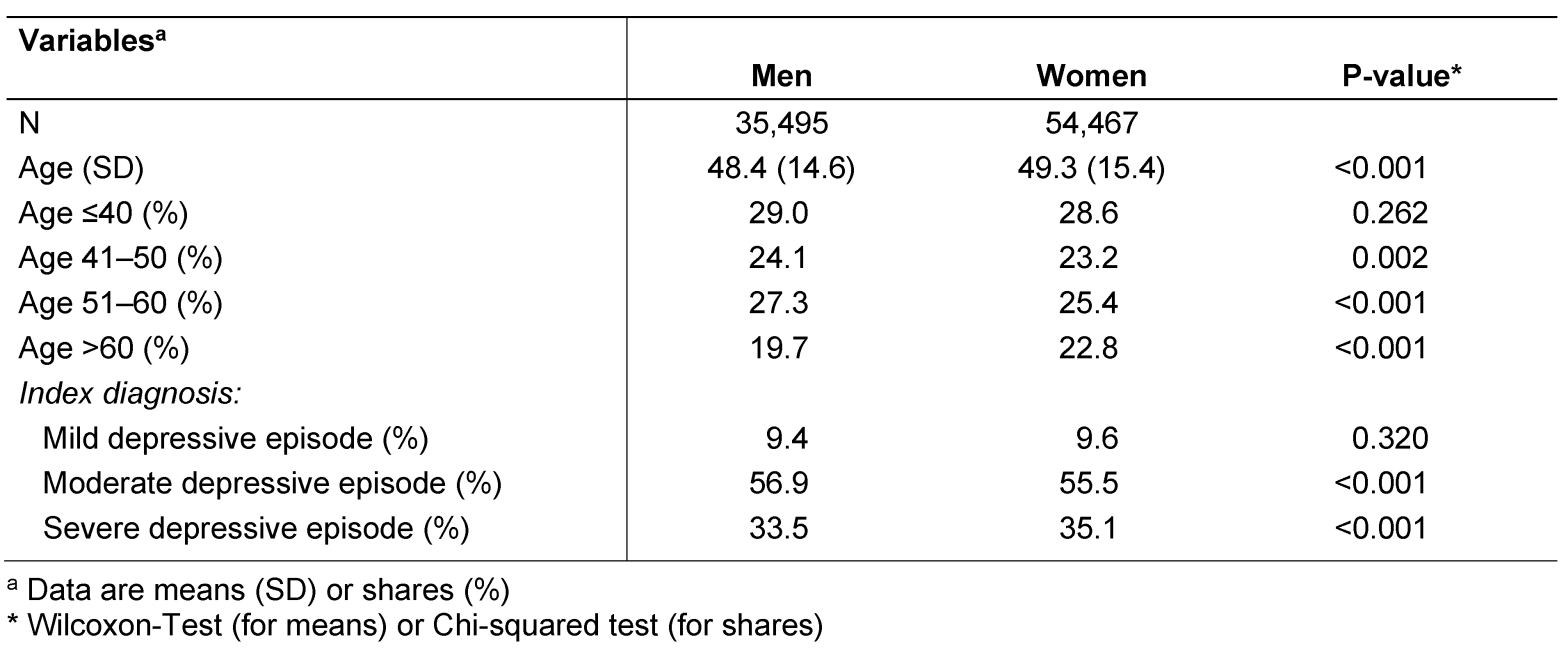
Baseline characteristics of men and women diagnosed with depression in neuropsychiatric practices

**Table 2:**
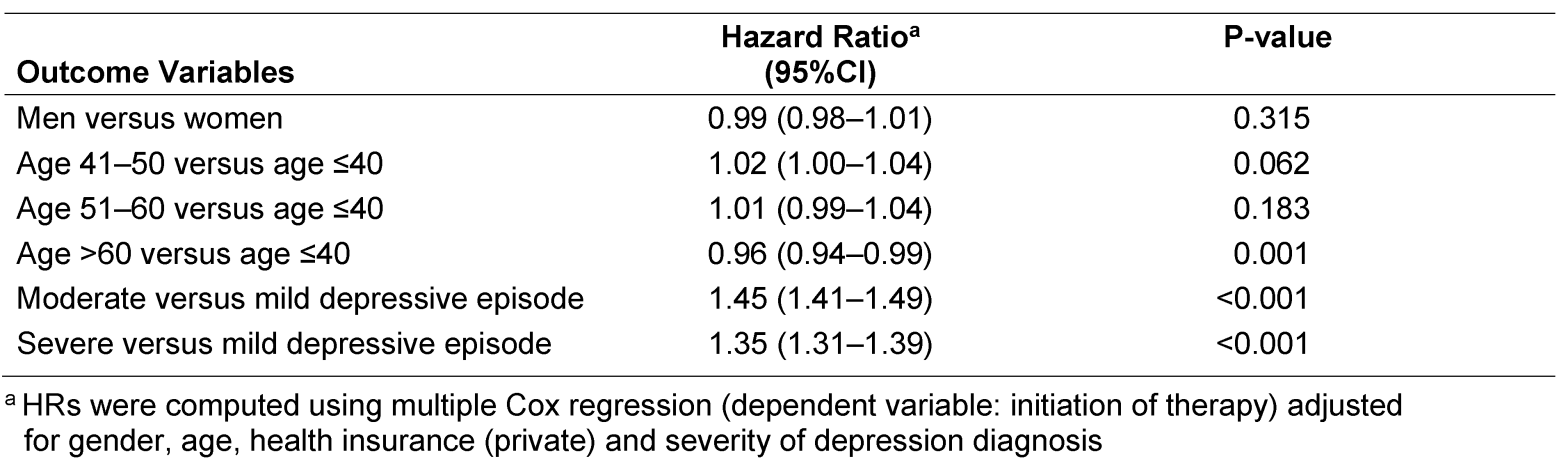
Patient characteristics as predictors of treatment initiation: multivariate Cox regression model

**Table 3:**
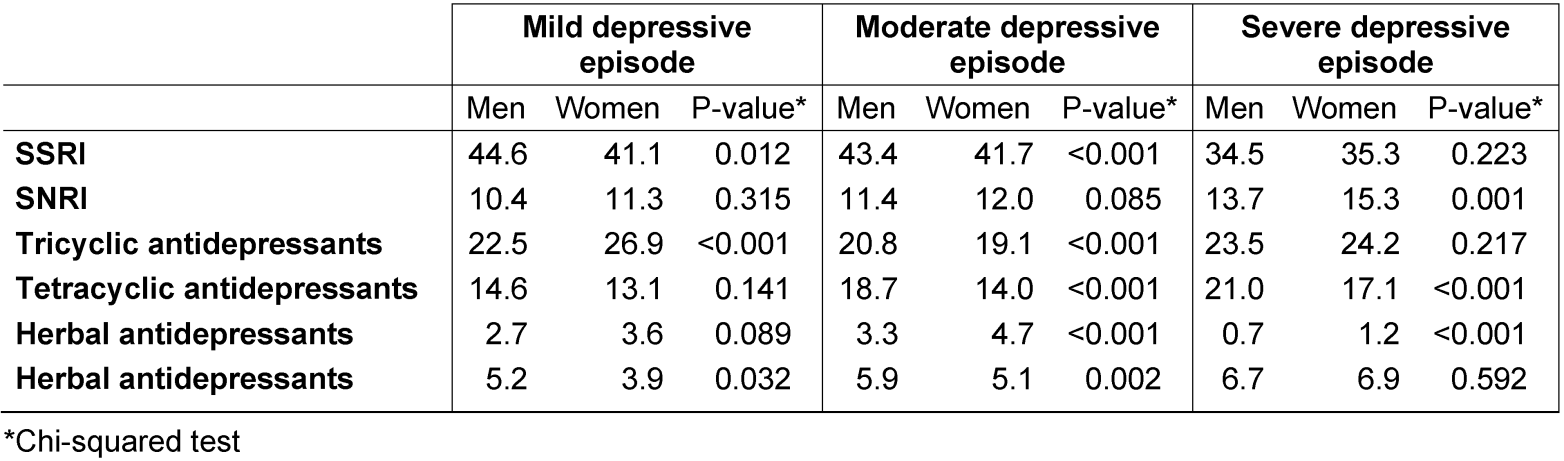
Antidepressant classes initially prescribed in men and women diagnosed with depression in German neuropsychiatric practices
